# The One Nutrition in Complex Environments (ONCE) study protocol: a cluster-randomized multi-level multi-sectoral intervention to improve nutrition in Uganda

**DOI:** 10.1186/s13063-022-06170-7

**Published:** 2022-04-01

**Authors:** Stacy P. Griswold, Anastasia Marshak, Merry Fitzpatrick, Daniele Lantagne, Kate Shoenmakers, Marlene Hebie, Anne Radday, Hugo De Groote, Saurabh Mehta, Greg Gottlieb, Patrick Webb, Shibani Ghosh

**Affiliations:** 1 Friedman School of Nutrition Science and Policy, Tufts University, Boston, MA USA; 2 Feinstein International Center, Tufts University, Boston, MA USA; 3 Department of Civil and Environmental Engineering, School of Engineering, Tufts University, Medford, MA USA; 4 GOAL International, Dublin, Ireland; 5 International Maize and Wheat Improvement Centre (CIMMYT), Nairobi, Kenya; 6 Division of Nutritional Sciences, Cornell University, Ithaca, NY USA

**Keywords:** Nutrition social behavior change, Cluster-randomized trial, Agriculture, Water

## Abstract

**Objective:**

To implement and measure the effects of a multi-level multi-sectoral social behavior change (SBC) intervention in Agago District of Northern Uganda and to determine the potential for scale-up.

**Intervention:**

Compare the Nutrition Impact and Positive Practice (NIPP) approach to a NIPP+ approach. The NIPP approach involves nutrition education and SBC, whereas the NIPP+ adds agricultural inputs, training, and tools to support improved farm and water quality practices. The intervention effect will be measured through lower levels of aflatoxin in grain, lower water contamination, and improved knowledge on nutrition and health.

**Design:**

This is a three-arm cluster-randomized controlled superiority trial (cRCT). The study arms include the following: group 1: NIPP; group 2: NIPP+, and group 3: control. Groups 1 and 2 will receive a 12-week intervention (NIPP or NIPP+) with active monitoring and longitudinal follow-up at 2, 6, and 12 months post-intervention. Additionally, an in-depth process and performance evaluation of each intervention arm will be undertaken using quantitative and qualitative methods. A total of 60 clusters from 5 subcounties of the Agago district will be randomly selected, and 15 households per cluster will be recruited using specific inclusion/exclusion criteria for a total of 900 households (300/arm). Respondents for the qualitative portion will be purposely selected.

**Analysis:**

We will collect data from all participants at 3 time points: baseline, endline, and 12 months post-intervention. The analysis will utilize intent-to-treat (ITT) using the initial randomization of the treatment arms to determine the overall impact of having the NIPP vs. NIPP+ vs. control. Mixed effects models will be used to determine the role of time-variant and invariant individual household, and community characteristics, as well as measures of exposure and integration on key outcome indicators. A difference-in-difference analysis (baseline/endline, baseline/12 months post-intervention, endline/12 months post-intervention) will also be used to triangulate findings.

**Trial registration:**

NCT04209569. One Nutrition in Complex Environments (ONCE) Registered 24 December 2019

**Supplementary Information:**

The online version contains supplementary material available at 10.1186/s13063-022-06170-7.

## Introduction

Globally, while child stunting has been declining, the prevalence of severe and moderate acute malnutrition has remained high and resistant to change. Much of the burden of wasting incidence and prevalence resides in complex environments, such as those in Northern Uganda, characterized by weak infrastructure, poverty, and a history of protracted conflict. Furthermore, while stunting and wasting are considered as separate phenomena, they often occur within the same population and/or the same individual. Research suggests that the underlying drivers of risk of developing wasting are similar to those of stunting [1–5]. One systematic review found significant overlap in drivers of stunting and wasting including maternal education, age and gender of the child, household wealth, prolonged duration of breastfeeding (> 12 months), low birth weight, maternal age (< 20 years), source of drinking water, maternal BMI (< 18.5), diarrhea, father’s education and place of residence (rural) [6]. Kikafunda et al. found that both poor health access and fuel access were simultaneously correlated with both stunting and wasting while Vella et al. identified diarrhea as a risk factor for both stunting and wasting in Uganda [7, 8]. While there are similar risk factors for a child to become wasted or stunted during both infancy and childhood, those same risk factors may vary in intensity and timing [9]. The rates of malnutrition (both globally and in Uganda) make a compelling case for interventions that, as well as improving the nutritional status through nutrition-specific interventions, also attempt to prevent the occurrence of malnutrition in the first instance through complementary nutrition-sensitive approaches in the form of multi-sectoral interventions.

Evidence of the effectiveness of the integration of nutrition-specific and nutrition-sensitive social behavior change (SBC), particularly in the areas of water, sanitation, and hygiene (WASH), is mixed. In the WASH Benefits study conducted in Bangladesh, a seven-arm cluster-randomized study of pregnant women and their offspring, integrating traditional WASH approaches (e.g. chlorinated drinking water, handwashing, upgraded sanitation at household level) with nutrition-specific interventions (provision of counseling on infant nutrition and exclusive breastfeeding and provision of lipid-based nutrient supplements to the infants starting at 6 months of age). The results indicated no impact on linear growth at 24 months of age between integrated and un-integrated intervention arms [10]. Apart from linear growth, however, the study’s WASH approaches (either alone or combined with nutrition) lowered the 7-day diarrhea prevalence, except the provision of chlorinated water. This could imply that WASH interventions add no added-value compared to nutrition-specific interventions; however, a systematic review of WASH interventions found modest improvements in linear growth among children exposed to interventions targeted at improving water quality as well as improved sanitation facilities [11].

Community-based interventions to decrease contamination of communities’ water sources include building improved sources (e.g., boreholes) and encouraging community members to gather water from these improved sources [12]. However, household preferences for unimproved water sources like rivers whether out of convenience (closer to the home) or habit (gathering point for community members) may lead to higher rates of microbial infections [12–14]. Even when households collect water from boreholes, microbial contamination is likely because of transportation, storage practices, and household water management habits [15, 16]. Water chain management from source to point of use, community management of human and animal fecal matter, and individual household hygiene practices all impact contamination levels of households’ drinking water and are linked to children’s nutritional outcomes including increasing the risk of relapse into acute malnutrition after nutritional rehabilitation [13, 17, 18]. A sub-study of the Uganda birth cohort reported the potential for point of use (i.e., household-based) water quality improvement interventions in decreasing *E. coli* contamination of household drinking water, thus decreasing the severity of environmental enteric dysfunction (EED: an inflammatory process linked to poor health and hygiene) and improving growth in infants aged 12–16 months [19].

Both improved household food production and improved income coupled with SBC messaging are postulated as actions that can support improved household food security and nutrition in rural households [20]. However, most studies reviewed in Masset et al. were too methodologically weak to support the above noted hypothesis [20]. Since then, significant emphasis has been placed on understanding the efficacy and effectiveness of agricultural innovations such as homestead gardening, interventions targeting improving access to animal source foods, and micronutrient biofortified staples on nutrition outcomes [21]. This is a crucial move in the right direction, but we still need to extend thinking around nutrition-sensitive agriculture to understand the intersection of the seemingly disparate elements of SBC, nutrient-dense food production, health, food safety, and agricultural growth to promote income growth. For instance, although animal source foods are known to promote linear growth, the presence of livestock within homes could increase the risk of wasting, linking improvements in livestock rearing with degradation in WASH practices and declination in nutritional status [22, 23]. Livestock are rarely considered in messaging around hygiene behavior with the primary focus on human rather than animal feces [22]. These findings emphasize the importance of linking food/dietary quality interventions to WASH, animal health, and human-animal interaction interventions.

Furthermore, while there has been significant development of agricultural and/or livestock technologies including new crop varieties, improved breeds of livestock, management practices for reducing post-harvest loss among others, the adoption and uptake of these technologies may be limited [24]. In the Agago district in Northern Uganda, only 19% of 600 interviewed households used improved seeds and 3% used agrochemicals (fertilizers). Only 11% of the interviewed households in Agago had improved crop drying techniques, 18% practiced improved post-harvest storage, and 13% used improved marketing strategies [24]. These are significant concerns to a household or community’s health since poor crop management and post-harvest practices are significantly associated with high levels of aflatoxin contamination in maize-producing countries such as Ghana with about 30% of loss of crops such as maize due to poor moisture management [25]. In Uganda, aflatoxin exposure in turn has been linked to premature births, low birth length and weight, all factors affecting the first 1000 days [19, 26, 27].

One strategy to support the uptake of technologies and improved practices could be through supporting self-assessment, e.g., assessment of one’s own grain for moisture and water for microbial quality. There exist innovations for such self-assessments that have been field tested. For instance, Bain et al. identified 44 water quality tests that have been used and validated in field-based assessments [28]. They assessed the suitability of each test including the cost and found all easy to use, with 18 tests yielding a presence/absence result while 26 provide enumeration of the bacterial concentration. The Aquagenx water quality system is one such kit. This kit has been validated in the Uganda panel and birth cohort study of the Nutrition Innovation Lab [19]. Assessment and management of moisture in crops, particularly maize, has been extensively studied. Battery-operated hygrometers can be purchased for about $0.90 per unit through an online reseller. The device is reusable, can detect moisture levels in maize within 15 min, and allows farmers and traders to determine whether maize is dry enough to be safely stored for later human consumption [29].

A recent study, analyzing policies over the last 20 years, found a shift towards increased integrated government action on nutrition over time, especially the 2011–2015 analysis period [30]. However, considerable variations exist across sectors and over time, and the sustainability of nutrition integration efforts remains contentious. A related study found that increased nutrition policy integration is explained by different factors, including promotion of issues and policies by international actors as well as by domestic policy entrepreneurs [31]. Specifically, within the Ugandan context, emphasis is being placed by the Government of Uganda towards the adoption of hybrid maize varieties to boost productivity and food security; however, these varieties are often susceptible to insects and loss which might serve to be a disincentive towards adoption. A randomized control trial on improved maize variety and improved storage technology adoption conducted in Uganda found that farmers were more likely to plant the hybrid maize varieties (than traditional lower yielding varieties) if they had access to improved storage technology (e.g., the Purdue Improved Crop Storage (PICS) bags). These small holders were also more likely to store maize for a longer time and reported a substantial (statistically significant) drop in storage losses. These small holders were also less likely to use storage chemicals than farmers who did not receive access to the technology [32]. PICS bags are trademarked hermetic bags that have two inner bags made of high-density polypropylene with an outer woven polypropylene bag. These bags were developed to reduce insect damage but have been successfully used to reduce the risk of aflatoxin contamination in maize [33, 34]. Within the context of water management and reducing contamination from pathogens at the community and household levels, the following approaches have been suggested: using different containers for transporting and storing water from different sources (especially potable vs. non-potable), digging separate traditional wells for animal and human use (ongoing work in Chad), control access to potable water sources by enclosing the source with available natural material, and importantly expanding key messages around hygiene to emphasize reducing exposure to animal feces as well as human feces [22, 35].

Major considerations in determining the potential of interventions to be scaled up within complex environments include the type of implementation and sustainability of that intervention after the end of project activities. While there is evidence of the importance of integration, most multi-sectoral interventions still take place in a stove-piped fashion with activities being co-located rather than integrated both spatially (all activities in the same communities) and sectoral (the elements of action being mutually supportive). There are clearly successes in the treatment of malnutrition (with some concerns of sustainability) but there is very little understanding on how behavior change will translate into action, thereby preventing the onset of either wasting or stunting. This results in lost opportunities to (i) identify and act on potential synergies, (ii) address possible negative externalities, and (iii) build up household awareness of and ability to address underlying problems to prevent the cycle of malnutrition.

Modifications to, and integration of, existing interventions, rather than a completely new set of activities, can adapt these activities to complex environments. When combined with ongoing research to catalyze positive change, they could result in the rapid, cost-effective, and sustained reduction of nutritional vulnerabilities in the face of multiple shocks. Furthermore, to be effective, interventions must engender change that persists beyond the intervention and presence of external actors. A recent study on the sustainability of food assistance projects for the USAID Food and Nutrition Technical Assistance III Project (FANTA) found that evidence of impact at the end of a project period did not necessarily persist 2 years later [36]. Post-project completion, there was evidence of “dis-adoption” of key behaviors promoted through SBC, such as continued feeding during episodes of diarrhea, lack of timely introduction of complementary feeding, and reduced handwashing. Of particular relevance for this study is the finding that when water quality testing was done by external actors during a project, community water committees did not assume responsibility for that task after program completion [36].

### Aims and hypotheses

This study asks the research question “Does enabling families (particularly mothers and other caregivers) to ‘assess and act’ on drivers of malnutrition through a targeted SBC+ (NIPP+) package succeed in a sustained reduction of risk factors thereby improving child health and nutrition?” We hypothesize that a multi-level multi-sectoral “value added” intervention package will enable mothers and other caregivers to “assess and act” on drivers of child malnutrition (e.g., stunting and wasting) in a sustained, scalable manner. The package involves catalysts for change (i.e., behavior change messages for improved practices coupled with innovative tools for households) to encourage action at the household level on these drivers (poor-quality diets, poor health, poor water quality at household and community, aflatoxin contamination of household crops). The goal is to enhance their problem awareness and their own ability to improve behaviors and practices. This model of “facilitated transfer of responsibility” at different levels (household/farm and community) offers huge potential for success at scale in complex environments where physical access, quality of services, and functional markets are all constrained. To test the above stated hypothesis, this study aims to implement and measure the effects of a multi-level multi-sectoral intervention in Northern Uganda and determine the potential for scale-up in a complex environment. The specific aims of this intervention study are to:

Aim 1: Implement and test an SBC program (NIPP) alone (free-standing) and in combination with a “value-added” approach (NIPP+). The latter includes access to innovative low-cost tools and technologies relating to WASH and agriculture.

Aim 2: Identify best practices emerging from the implementation of both approaches through process and program monitoring for effective integration, implementation, scale-up, and uptake of multi-sectoral and multi-level packages in complex environments to ascertain potential for scale-up.

Aim 3: Study the sustained impact of the NIPP approach and the “value added” package (NIPP+) on knowledge and practices by gender within participating households, environmental risk factors, child health, and nutritional status through a rigorous impact evaluation and longitudinal monitoring system.

## Study overview, design, and setting

This is a three-arm cluster-randomized controlled superiority study (cRCT) with a 1:1:1 allocation ratio. The study arms will be group 1: NIPP arm, group 2: NIPP+ arm, and group 3: control arm. Each intervention arm will receive a 12-week intervention (NIPP or NIPP+) with active monitoring and longitudinal follow-ups post-intervention.

The study will be conducted in the Agago District of Northern Uganda in communities in which the subjects live and work. Agago District is an example of a complex environment in that households and communities there are recovering from conflict and are also likely to suffer from frequent droughts, livestock and human disease, and other hazards. Subcounties included in the study are detailed in Table 1. Exclusion criteria for subcounties were as follows:

- Areas with similar behavioral changed interventions like Community Conversation (CC) and Community Led Total Sanitation
- Urban or semi-urban areas
- High levels of food insecurity
- Areas of current and previous nutritional interventions

**Table 1:** List of subcounties from Agago District, Uganda, and maximum number of clusters possible per subcounty

Subcounty^a^	Number of parishes	Number of households in subcounty (estimated)	Max number of clusters^b^ in any one subcounty
Omot	4	1342	12
Lokole	7	2398	21
Omiya Pachwa	6	3226	18
Patongo	6	3239	18
Arum	8	3993	24
Kuywee	5	2874	15
Parabongo	7	2015	21
Lira-Palwo	3	2070	9
Paimol	4	2172	12
Agengo	6	2791	18

^a^Subcounties were excluded based upon being a locale with similar behavioral change interventions, containing an urban or semi-urban area, having high levels of food insecurity, or being included in current and previous nutritional interventions

^b^A cluster will be a group of 3 NIPP (or NIPP+) circles whether be the women’s, men’s, or community circles and be made of one or more “villages” because of the small population size in some of the parishes of Agago

The nutrition impact in positive practice (NIPP) approach developed by GOAL Global is a gendered, grassroots SBC approach, directly tackling a package of the underlying behavioral determinants of malnutrition, irrespective of the specific manifestation. The approach targets factors that are both nutrition-specific and nutrition-sensitive. It is also an intervention that has a strong monitoring, evaluation, and adaptive learning component built in. The NIPP approach involves the creation of community, male, and female circles in each community with messaging and activities targeted towards the three circle groups in varying intensities. While the male and female circles meet 2–3 times per week for a maximum period of 12 weeks, the community circles will meet for approximately 3 h at any one time over a period of 2–7 days. The aim of the shorter community circle exposure is to introduce the core topics and behaviors that will be further reinforced in both the female and male circles. Each circle is facilitated by a trained positive deviant NIPP volunteer (community, male and female volunteers), who is supported by the NIPP program staff (nutrition officers). While the female circles undertake all the practical activities, the male circles focus on ensuring that male household members understand and support female household members to make positive changes for their families, while being actively encouraged to participate in the training sessions. The NIPP approach targets entire clusters and often implements multiple circles within a cluster (for example, an entire district or administrative area); for the purpose of the study, a “cluster” will be a group of 3 NIPP or NIPP+ circles whether women’s, men’s, or community circles. A “cluster” in the Agago District context will be made of one or more “villages” because of the small population size in some of the parishes of Agago (see Table 1).^1^

### Study groups

*Group 1: standard NIPP approach*, the 12-week lesson plan for the standard NIPP circles are divided into 3 components: (i) hands-on behavior change sessions focusing on the key previously identified causes of malnutrition to improve awareness and practice, (ii) micro-gardening for improved household nutrition security, and (iii) participatory cooking demonstrations to stimulate improvements in nutritional status and care practices.

*Component 1: Practical behavior change communication and counseling* includes four standard activities: (1) fabrication and use of local handwashing facilities with soap or ash, (2) fabrication and use of simple latrines using local materials, (3) fabrication and use of fuel-efficient stoves, and (4) practical demonstrations on food processing, preservation, and storage. The component also includes up to eight topics that are developed and implemented based on the findings of the formative research and barrier analysis and selected based on community-identified need and interest. In this study, those topics included improving access to and knowledge of insecticide-treated bed nets, improved drinking water storage practices, time-saving tips to improve quality of complementary meals, need to improve storage facilities for grain, and improving the enabling environment to support exclusive breastfeeding.

*Component 2: Micro-gardening for improved household nutrition security* promotes nutrition security through dietary diversification at the household level through practical learning around the construction and maintenance of a small-scale garden and the provision of “starter” seed packs containing eggplant, sukuma (collard greens), and *Amaranthus*. To ensure that proceeds from the gardens produce nutritional benefits at the household level, consumption of locally available food is reinforced through behavior change sessions and cooking demonstration sessions. Participants are also encouraged to sell any surplus production for income generation.

*Component 3: Participatory cooking demonstrations:* complimenting component 2, participants will be provided lessons illustrating the affordability of a diverse and nutritious diet using locally available food to improve the nutritional status of their household. An interactive peer-guided cooking demonstration session is used to teach caregivers how to diversify the household diet using recipes made of locally available food with high nutritional value that includes home-grown, market, or wild foods. Up to six high-energy and nutritious recipes are taught to the participants, then repeatedly demonstrated over the course of the 12 weeks (see Supplementary Materials for example recipes).

*Group 2: the NIPP+ intervention*, in addition to the three components of group 1, NIPP+ participants will receive access to innovations enabling and encouraging households and communities to translate knowledge into positive practices. The innovations and access to vendors who sell innovations will be made available during the training to the NIPP+ volunteers who will facilitate trainings and encourage access to vendors during the circle meetings. Most of the additions will be made accessible at a subsidized/low cost.^2^ Two additional components will be added to the NIPP curriculum:

Component 4: Pre-harvest and post-harvest agricultural practices: training on methods/techniques to be used pre-harvest in the selection of appropriate maize seed varieties to ensure that the maize is of good quality, drought resistant, and less likely to suffer from insect or disease damage, good agronomic practices to maximize yield and minimize crop loss, appropriate use of fertilizer, harvesting, and post-harvest storage and drying methods to ensure moisture levels below 13.5%. The tools that will be made accessible through vendors and the NIPP+ volunteers include:

1. *Hygrometers*^3^: standard devices that measure humidity and temperature of produce stored in home settings developed through a collaboration between Purdue University, CIMMYT, KALRO, and USAID. These devices measure the relative humidity in the air. When placed in a hermetic bag with grain, the reading will inform the farmer if the air inside (and the grain by proxy) is too wet (i.e., if greater than 65% humidity) or at an acceptable level to prevent mold growth. Using hygrometers ensures that maize is properly dried before storage. To introduce the hygrometers to participants, a practical session in which grain samples that have been exposed to different levels of moisture are placed in a storage bag and the hygrometer is used to measure the humidity. This will be integrated into the module detailing proper drying techniques after shelling, using a tarp.
2. *Hermetic bags:* portable and reusable 50-kg storage bags made from thick polyethylene that restrict gas exchanges such that the oxygen in the bag is replaced by carbon dioxide through natural processes in the maize. This process kills insects and stops fungal growth and the production of aflatoxins in the stored grain. At least one bag will be provided for free to every household, and more will also be made available through market vendors.
3. *Improved seed varieties:* maize varieties tolerant to different stresses (drought, low soil fertility, and disease) have been developed and tested widely in partnership with the National Agricultural Research Organization (NARO) in different parts of Uganda, including northern Uganda. These new stress-tolerant maize varieties are high yielding and are important in improving maize productivity at the farm level through enhanced yields and resilience to various abiotic and biotic stresses commonly encountered by farmers. They are produced by local seed companies and distributed through private-sector agro-dealers.
4. *Training in good agronomic practices (GAP):* Integrated soil fertility management (ISFM) practices will be implemented to increase crop productivity in a profitable and environmentally friendly way. These practices include (1) the combined use of organic manure and mineral fertilizers and (2) dual-purpose legume-cereal rotations and inter-cropping. Inter-cropping and crop rotations help in preventing pests and diseases to spread throughout the field, increase soil fertility, especially in the case of legumes, help in controlling weeds, and will be adapted to the local conditions/farming context. Farmers will be trained on improved maize varieties and good agronomic practices during on-farm demonstrations.

*Component 5: Water quality assessment and management (household and community level):* training on methods/techniques and doable actions^4^ for improving WASH practices to be used in conjunction with assessment of water quality. This component will rely on a resource pack that has been developed by the Government of Uganda as part of the Nutrition Assessment, Counselling, and Support (NACS) approach to facilitate training of village health workers, community knowledge workers, peer support groups, and other outreach workers. This will include training on how to keep the water source safe, practice doable actions like handwashing, creating tippy taps, using AquaGenX water testing kits, methods for separating animals from humans to reduce fecal contamination, use of a jerry can to safely store and keep water purified after boiling, using chlorine tablets, or solar disinfection methods. These modules are delivered by trained volunteers at a mutually agreed location within the community.

*Group 3: Control*, this group will receive no intervention and will serve as a control group for the intervention assessment***.***

### Intervention preparation

#### NIPP and NIPP+ intervention training/session modules

Through the 12-week intervention period, NIPP volunteers will conduct sessions that will cover the following elements to the circle participants:

1. Promotion of food hygiene practices;
2. How to keep the water source safe by identifying the small doable actions that will be used as a metric of assessment such as:
  - Using different containers for transportation and storage of household potable water;
  - Regular cleaning of storage and transportation water containers;
  - Handwashing and use of tippy taps to conserve water;
  - Separate animals from humans within the household to reduce fecal contamination of water;
  - Use of simple water purification methods (e.g., boiling, solar disinfection, etc.) (NIPP+ only);
  - Demonstrate how simple water quality kits can identify contamination (NIPP+ only); and
  - Demonstrate simple methods (at the household and community levels) to support improving the quality of water (Hamoudi, 2012) (NIPP+ only).

To reinforce impact and empower the women and men in the NIPP circles, we will encourage them to maintain a record of their results on forms adapted for the general literacy levels of the target populations. All materials will be tested and contextualized to the local situation and translated into Acholi.

Agricultural demonstration plots will be planted and used to train the NIPP+ volunteers on GAP. The harvest from the demonstration plots will be bagged in both hermetic and non-hermetic storage bags to use for demonstration at the NIPP+ volunteer training. We anticipate providing hermetic bags, seed packs, and fertilizer samples to the NIPP+ volunteers who will demonstrate the use of these inputs within their own circles. Volunteers will be encouraged to store their maize from the harvest of the demonstration plots in hermetic bags to show the circle participants the benefits of using the bag.

In addition, inputs provided to the NIPP+ volunteers to improve the household’s WASH environment will include Aquagenx CBT H_2_S water testing kits (CBT kit; Aquagenx, Chapel Hill, NC) for demonstration purposes and 5-L jerry cans. NIPP+ volunteers will be trained to use the water quality kits and then asked to demonstrate the kit to their NIPP+ participants along with clear doable actions to improve WASH-specific behaviors. To ensure that NIPP+ volunteers have access to seeds, hygrometers, hermetic bags, and water quality testing kits (so that participants might buy them at low cost), participants and volunteers will be linked to agro-dealers who sell seeds of improved maize varieties, hermetic bags, and moisture meters and to vendors for water quality kits. Though we will aim to make these available for low cost, it may be that the kits will have to be provided (at least one kit) per household to ensure households are able to try out the kits to better understand their value. For both NIPP and NIPP+, pilot high-energy nutrient-rich recipes will be developed through market surveys and observation visits to local homesteads to determine the full range of foods available, accessible, and affordable and then use the results of the market surveys to pilot a variety of high-energy nutrient-rich recipes.

The identified volunteers in both NIPP and NIPP+ groups will undergo a rigorous training in two phases — a preliminary phase whereby all volunteers assume the role of the circle participants and are trained by project staff in collaboration with relevant district government staff and a second phase whereby the volunteers become the circle lead and practice leading sessions themselves.

#### COVID-19 reconfiguration

To accommodate safety measures put in place to mitigate the potential spread of COVID-19, both the NIPP and NIPP+ modules were altered to add a session about COVID-19 which included practice on how to properly wear a mask, sensitization about what COVID-19 is, symptoms, and how it is spread between individuals, and additional emphasis on the importance of handwashing with soap or ash to prevent the spread of disease. In addition, each session was amended to begin with a demonstration of proper facemask protocol so that all participants were wearing facemasks correctly for the duration of the program. These additions expanded the standard program design from 12 to 16 weeks.

### Data collection

**Fig. 1:**
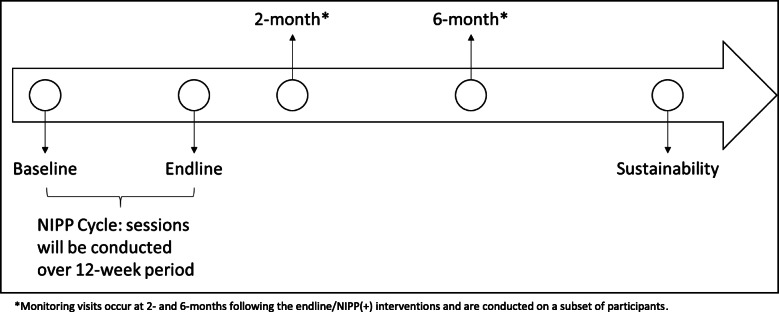
Intervention timeline and key milestones

Data will be collected on all participants (*n* = 900 in 60 clusters) at the start of the program (baseline), at 4 months after baseline (endline), and at the sustainability measurement (12 months after the end of the intervention) (Fig. 1) by enumerators trained in the proper anthropometric, survey, water collection, and maize collection methods. We will also collect data on a subset of households 2 months and 6 months after the completion of the intervention (“Monitoring Activity” in Fig. 1). We will collect maize samples a few months after endline and a few months after sustainability, which corresponds approximately to September in the intervention year and September in the follow-up year following each year’s harvest. Finally, samples using an in-development smartphone technology for assessing aflatoxin levels in capillary blood will be collected in parallel with the maize aflatoxin data collection (see the “Tools and data collection” section for more detail).

To assess the potential for scale and identify best practices, qualitative assessments will be conducted at the end of the intervention (endline), at follow-up 2 months and 6 months, and as part of the sustainability assessment (12 months after the end of the intervention). The format used for the qualitative assessment will be structured key informant interviews for program staff and NIPP and NIPP+ volunteers along with focus group discussions with NIPP circle participants. The planned data collection for qualitative assessments are:

1. Endline: Structured key informant interviews with program staff and NIPP and NIPP+ volunteers facilitating the circles. The focus will be discussing difficulties they faced during planning and implementation, how they worked through those difficulties, the relative success of different approaches (both NIPP and NIPP+), and lessons learned from that process.
2. Endline-sustainability: Focus group discussions of 30 female and 30 male participants will be interviewed to understand what motivated those who decided to invest in the innovations, or to adopt promoted practices, as well as what discouraged other participants from doing the same.
3. Two- and 6-month follow-up and sustainability: Focus group discussions with the same 30 female and 30 male participants to assess any shift in the various practices and better understand factors that encouraged or discouraged them in maintaining the innovations or practices, as well as their perceived costs and benefits.

### Sample size calculation and sampling strategy

To detect meaningful differences in the change in the primary outcome measures across the three arms with a power of 0.80, a significance level of 0.05, and an intra-class correlation of 0.05, each treatment arm will require 17 clusters with 15 households (with children under the age of 2) in each cluster (Table 2). We used secondary data on all outcome measures (except total coliform) from Northern Uganda and collected as part of the Feed the Future Innovation Lab for Nutrition Uganda Birth Cohort Study [24]. We aim to follow the same households throughout the duration of the study. Thus, the sample size is adjusted for 20% attrition. This increase will come from additional clusters and not households (to keep the integrity of the NIPP 15 households/circle programmatic design). The sample will include 20 clusters per arm for a total of 60 clusters and 15 households per cluster with an overall household sample size of 900. Based on the results of cluster randomization, trained study personnel will approach households that meet the study’s inclusion criteria in the parishes selected for the study. They will approach eligible households and seek consent until a total of 15 households agree thus forming a cluster. The sampling at the household level is purposeful. A random subsample of households will be taken at 2 months and interviewed again at 6 months post-intervention. This sample size is a fixed sample of 82 households per intervention arm.

**Table 2:** Sample size calculation for quantitative evaluation

Category	Mean	*SD*	ICC^a^	Effect size (Cohn’s)	Sample size/treatment arm	Total sample size
*E. coli*	31.56	45.14	0.05	0.67	120 (20 clusters*5 hh per cluster + 20% for attrition)	≈ 360
Aflatoxin	20	43.6	0.05	0.34	306 (17 clusters*15 hh per cluster + 20% for attrition)	≈ 900

^a^The ICC calculated for aflatoxin for mothers was close to 0 but came from a small sample size. Thus, we have decided to overestimate the ICC to 0.05

The criteria for community selection include food security, water access, security, community cohesion, and geographic proximity to one another to decrease the probability of contamination between the arms (GOAL 2018). These criteria will be applied in the selection of all 60 clusters (intervention and control). Tufts will utilize block randomization to randomly assign clusters to 1 of 3 treatment groups and will only share the list of clusters by treatment arm (not how they were assigned). A total of 60 clusters (a “cluster” is a group of 3 NIPP circles and may be at either parish or village level, dependent on population density — see Table 1 for subcounty-level estimates of cluster representation) will be randomly assigned to the three groups (20 clusters per group). We will randomize at the parish level by assigning random numbers to each parish, ranking the parishes by random assignment, and assigning to a treatment group after the ranking is complete such that group 1 is randomized to Control, group 2 to NIPP, and group 3 to NIPP+. The households will be selected purposefully after clusters have been randomized to specific arms. Each cluster in groups 1 and 2 will have one male, one female, and one community NIPP circle. Each circle will include 10–15 individuals with the cluster serving as the primary unit of analysis. As this is a community-based intervention cluster-randomized trial, it is impossible to conceal from participants which treatment arm they have been randomized to and which intervention they have the opportunity to consent to being a part of.

### Inclusion/exclusion criteria

In groups 1 and 2, within each cluster, community leaders will be approached to identify literate individuals with specific practices that are recommended by the NIPP approach. These individuals will be invited to become NIPP volunteers. The inclusion criteria for the NIPP volunteers are as follows: willingness to implement the interventions in the circles over a 12-week period, have a healthy child age less than 24 months, and are approved by community leaders. The inclusion criteria for the NIPP female circle participants are as follows: willingness to participate in the group, are planning to stay in the area for 12 weeks, and have a child under the age of 2 (irrespective of nutritional status). The inclusion criteria for the NIPP male circle participants are as follows: willingness to participate, are planning to stay in the area for 12 weeks, and are a spouse, brother, and/or other influential male family members of the female caregiver enrolled in the female circle. There were no specific criteria for the community circle other than being key community leaders. Age is not a direct criterion either for the NIPP volunteers or for the NIPP circle participants though we specifically note that the participant must be a caregiver of a child under the age of 2. In the context of Uganda that caregiver could be a grandmother, adoptive parent, or older sibling, hence, there was no age cut-off. Exclusion criteria for both the NIPP and NIPP+ interventions are as follows: no child under age 2 in the household or not being present in the selected communities during the intervention and data collection periods and/or not willing to participate through the entire 12-week cycle. People can refuse to participate in the interview but continue to participate in the program. There are no specific criteria for default/dropout in the NIPP approach. However, volunteers will visit households where participants have missed 3 consecutive visits to inquire about their participant status. Participants who voluntarily exit the NIPP or NIPP+ program will still be surveyed but may voluntarily decline to participate in the interview at each time point. However, volunteers will visit households where participants have missed a session to inquire about their participant status. If a participant has missed 2 consecutive sessions and cannot be traced or voluntarily exit, they are considered as a defaulter.

### Primary and secondary outcomes

Please see Table 3 for a list of primary and secondary outcomes including the specific measurement and the time point of the measurement. We posit that implementing the NIPP+ approach translates into changes in knowledge and behaviors along with the ability to self-test that result in:

- Improved food safety and water quality through the reduction of environmental risk factors as measured by:
  - Reduced contamination of domestic water within households
  - Reduced aflatoxin contamination of common staple foods
  - Reduced aflatoxin levels in women and children (blood and urine levels)
- Improved nutrition and food security through improved household and individual diets and food security as measured by:
  - Improved dietary diversity in women and children
  - Improved household food security

**Table 3:** Primary and secondary outcomes

Category	Measure	Time point of measurement	Type of data collection
**Primary outcome measure**			
Nutrition	Women’s dietary diversity Infant and young child dietary diversity	Baseline, endline, 2- and 6-month follow-up, and sustainability	Quantitative
Water quality	*E. coli* (colonies/100 ml) at household and community water source	Baseline, endline, sustainability	Quantitative
	*E. coli* at household and community water		
Food safety	Aflatoxin levels in grain samples Aflatoxin levels (urinary and finger prick)	Endline, sustainability	Quantitative
Food security	Months of adequate household food provisioning, food security access scale	Baseline, endline, and sustainability	Quantitative
**Secondary outcome measures**			
Anthropometry	Child (6–59 months) weight for height *z*-score	Baseline, 2- and 6-month^**.**^ follow-up, endline, sustainability	Quantitative
	Child (6–59 months) weight for age *z*-score		
	Child (6–59 months) height for age *z*-score		
	Child mid-upper arm circumference (MUAC)		
Food security	Purchase and utilization of improved seeds	Baseline, endline, and sustainability	Quantitative
	Purchase and utilization of fertilizer		
	Maize yield		
	Purchase and use of hygrometers, tarps, and hermetic bags	Baseline, endline, and sustainability	Quantitative

The primary outcomes are a change/reduction in specific drivers (risk factors) of malnutrition. Primary outcomes are the outcomes of the greatest interest and are those that are going to be directly affected by the intervention (Vetter and Mascha 2017). Secondary outcomes are those that are affected through the change in primary outcomes. We posit that the identified primary outcomes will reduce malnutrition and improve the nutritional status of infants and young children. Secondary outcomes include child anthropometry (height-for-age, weight-for-age, weight-for-height), water quality (pathogens based on livestock composition along the water chain), and food security and agricultural practices (purchase and utilization of improved seeds, fertilizers, yield, purchase and use of hygrometers and hermetic bags). We do not measure anthropometry as a primary outcome as the goal is to improve (reduce) underlying risk factors. Furthermore, the sample size estimations are based on outcomes that reflect the reduction in risk factors and not reductions in malnutrition.

### Tools and data collection

#### Quantitative survey tool development

There will be two survey tools: a household survey and an agriculture survey. Components of the household survey are summarized in Table 4. The survey includes 18 modules that will assess basic demographic data of the household such as household composition and education status, food insecurity, 24-h diet recall of the female caregiver and index child, health information of the index child, a standard household possessions questionnaire, information on WASH practices and associated costs, fuel-efficient stove use, economic or social support provisions, garden use, knowledge of health behaviors, and a pre- and post-intervention standard metric survey. The following components will also be part of the household survey procedures:

**Table 4:** Program monitoring and assessment of sustainability

Measure (by treatment group)	Time point of measurement	Type of data collection
**Circle participation**		
Participation in meetings (based on ID confirmation of participants)	Monitoring through program (MEAL)	NIPP Booklet, in-home visit
# of meetings attended by participants per cluster	Monitoring through program (MEAL data)	NIPP Booklet, in-home visit
**BCC and counseling**		
Fabrication of handwashing facilities	Endline, 2- and 6-month follow-up, sustainability	Quantitative
Fabrication of simple latrines	Endline, 2- and 6-month follow-up, sustainability	Quantitative
Fabrication of fuel-efficient stoves	Endline, 2- and 6-month follow-up, sustainability	Quantitative
Continued use of handwashing facilities	2- and 6-month follow-up and sustainability	Quantitative
Continued use of latrines	2- and 6-month follow-up and sustainability	Quantitative
Continued use of fuel-efficient stoves	2- and 6-month follow-up and sustainability	Quantitative
Infants/young children achieving minimum acceptable diet	Endline, 2- and 6-month follow-up, sustainability	Quantitative
Newborns being exclusively breast fed	Endline, 2- and 6-month follow-up, sustainability	Quantitative
Newborns receiving complementary foods at 6 months	Endline, 2- and 6-month follow-up, sustainability	Quantitative
**Micro-gardening**		
Planted micro-garden	Endline	Quantitative
Continued maintenance of a micro-garden	2- and 6-month follow-up and sustainability	Quantitative
Using produce for household consumption	Endline, 2- and 6-month follow-up, sustainability	Quantitative
Selling produce from micro-garden	Endline, 2- and 6-month follow-up, sustainability	Quantitative
**Water quality**		
Reported knowledge of water quality assessment	Baseline, endline, 2- and 6-month follow-up sustainability	Quantitative
Reported purchase of water tests	Endline, 2- and 6-month follow-up, sustainability	Quantitative
Reported use of water tests	Endline, 2- and 6-month follow-up, sustainability	Quantitative
**Assessment of moisture**		
Reported knowledge of moisture assessment	Baseline, endline, and sustainability	
Reported purchase of hygrometers	Endline, 2- and 6-month follow-up, sustainability	Quantitative
Reported use of hygrometers	Endline, 2- and 6-month follow-up, sustainability	Quantitative
**Assessment of storage**		
Reported knowledge of improved storage	Baseline, endline, and sustainability	Quantitative
Reported purchase of hermetic storage bags	Endline, 2- and 6-month follow-up, sustainability	Quantitative
Reported use of hermetic storage bags	Endline, 2- and 6-month follow-up, sustainability	Quantitative
**Use of improved varieties and fertilizer**		
Reported purchase of improved maize varieties	Baseline, endline, and sustainability	Quantitative
Reported planting of improved maize varieties	Baseline, endline, and sustainability	Quantitative
Reported knowledge of vendors for improved varieties	Baseline, endline, and sustainability	Quantitative
# of interactions with vendors	Baseline, endline, and sustainability	Quantitative
Reported knowledge of fertilizer availability	Baseline, endline, and sustainability	Quantitative
Reported use of fertilizer	Baseline, endline, and sustainability	Quantitative
Reported use of improved seed varieties for the planting season after the intervention	Endline, 2- and 6-month follow-up, and sustainability	Quantitative

#### Anthropometry data collection

Children’s nutritional health status will be measured using standard anthropometric measures: mid-upper arm circumference (MUAC), weight, and height/length and presence of edema. Three measures of each indicator will be taken at the time points specified in Table 2. Female caregivers will also be assessed via anthropometry, specifically weight and MUAC. The enumerator training will be composed of a maximum 2-day training on anthropometry. At the end of the training, enumerators will be tested on the tool and their anthropometry skills. For the latter, we will use the Standardized Monitoring and Assessment of Relief and Transitions (SMART) approach where each enumerator will measure three to five children five times each. Given the importance of collecting accurate anthropometry data, in the field, enumerators will collect three measurements on height and weight for each child.

#### Blood or urine collection and aflatoxin assessment

Aflatoxin levels for the subsample of females and household’s eligible child/children or female caregivers will be assessed using finger-prick samples and/or 10 ml urine using field-friendly point-of-care diagnostic kits. These point-of-care diagnostics will be collected by trained enumerators or phlebotomists in collaboration with local government health systems and taken to a central makeshift lab suitable for diagnostic testing in field settings like Agogo. This method may also be used to test for aflatoxin contamination in the maize samples themselves along with the reference method.

#### Water sample collection for assessing *E. coli* and total coliforms

Microbiological water samples taken from the home and community will be processed within 6 h of sample collection using a membrane filtration method containing m-ColiBlue24 media (Hach Company, Loveland, CO). This method allows for the simultaneous analysis of *E. coli* and total coliforms. We will request each household to provide a 250-ml water sample from the storage container that is used for drinking purposes. At the community level, we will identify the common water sources and request the participants to provide a sample of 250 ml from each common water source. If households within a circle are using different water sources, we will collect a sample from each of the water sources used by the circle participants. For both household and community assessments, the sample will be transferred from a storage container to a sterile plastic bottle, placed into a cooler, and transported back to a makeshift lab where the water will be analyzed for *E. coli* and total coliforms. As we will only need 360 household samples per data collection (baseline, endline, sustainability), we will randomly select these households in advance in such a way that we are collecting 6 samples per every cluster (60 clusters × 6 samples = 360 samples). The same 360 households will be sampled at each time point. The community samples will be collected from every cluster.

At the lab, all water samples will undergo the following process: dilution with sterile buffered water, vacuum-filtered aseptically through 45-μm filters (EMD Millipore, Billerica, MA), placed in plastic Petri dishes with media-soaked pads, and incubated for 24 h at 35 °C. Two dilution volumes will be processed per water sample. As a standard quality control process, 10% of samples will be duplicated and 5% of samples will be blank/control. After incubation, colonies of bacteria will be counted, and concentrations calculated using the geometric mean of plate counts within a countable range (1–250 colonies). Plates with counts < 1 will be assigned 0.5 and > 250 were assigned 250.

As summarized in Table 4, the agriculture survey will be composed of 6 modules that capture information on crop diversity, agriculture inputs, crop yields and production, agriculture practices, and knowledge of GAP. In addition to capturing information through the survey, there will also be samples taken of harvested maize using the following method.

#### Method for sampling aflatoxin assessment in grain

The study team will collect grain samples from all households (*n* = 900). These will be analyzed for aflatoxins by International Maize and Wheat Improvement Centre (CIMMYT) at a makeshift lab established in Agago. Moisture levels in the staple crops will also be assessed on-site using existing moisture meters (from CIMMYT) that will allow the research team to corroborate the findings from the household monitoring using the hygrometers. For the aflatoxin assessment, grain will be collected in the home. If maize is stored in cobs, one cob of maize will be randomly selected from the home store at each assessment point The cobs will be hand shelled and thoroughly mixed to form a composite sample per household then dried on plastic mats to 14% moisture content. A 1-kg sample will be taken per household and transported to the laboratory for aflatoxin extraction and analysis. If the households have shelled the maize, samples will be taken from the storage bags using sampling spears, taking five samples per bag, thoroughly mixing the sample, and take a subsample of about 1 kg. Samples will be stored in a deep freezer (− 20 °C) until processing. For aflatoxin extraction, 50 g of maize flour will be transferred to a 250-ml flask, and 100 ml of 70% methanol (v/v 70 ml absolute methanol in 30 ml distilled water containing 0.5% w/v potassium chloride) will be added. The samples will be shaken for approximately 30 min at 300 rpm. The extract will be filtered through filter paper and diluted 1:10 in phosphate-buffered saline containing 500 μl/l Tween-20 (PBS–Tween). The extracts from the samples will be analyzed with an indirect competitive enzyme-linked immunosorbent assay (ELISA). Alternative low-cost options such as strip tests will also be explored.

#### Qualitative survey tool development

The tool being utilized for the qualitative assessment is developed on the basis of the intervention programming itself. A key informant interview guide has been prepared for the MEAL coordinator, the Nutrition Manager, and the Nutrition Officer (Program staff) while focus group discussion guides have been developed for the NIPP and NIPP+ volunteer and the circle participants. Qualitative data will be collected at endline with the MEAL coordinator, the Nutrition Program Officer, and 10 Nutrition Officers who work directly on the implementation of the NIPP and NIPP+ activities using semi-structured questionnaires. Interviews will also be conducted with 10 NIPP and 10 NIPP+ volunteers. Focus group discussions will be carried out with a maximum of 40 female participants (20 in NIPP and 20 in NIPP+ female circle) and 40 male participants (20 in NIPP and 20 NIPP+ circles). We anticipate each focus group to be composed of not more than 10 participants which will allow us to have 4 focus groups for women and 4 for men (2 in each intervention arm).

### Data management and statistical analysis

#### Data entry and management

To maintain a high level of data quality, the research team will use Digital Data Gathering (DDG) tools (which have become common in this field). The DDG reduces the number of man-made mistakes in the survey by providing a clear and easy-to-use platform for inputting data. In addition, almost real-time access (uploaded once Internet is available) to the data will allow a research to look for common mistakes (such as bundling of child age by 12, 24, 36, etc. months) and providing additional input/advice to the enumerators to eliminate those mistakes while still in the field. Once the data is collected, we will also look at whether certain responses are correlated to certain enumerators to make sure that the data was collected similarly across all enumerators. For the anthropometry data, values more than five standard deviations above and below the median WHO standards will be removed [3]. The final de-identified data will be shared via the Development Experience Clearinghouse (DEC).

#### Statistical methods for assessing impact

For the analysis, we will use an intent-to-treat (ITT) approach using the initial randomization of the treatment arms. While we know that there could be some contamination across groups or non-adherence, we will plan for the clusters to be sufficiently far apart that this should limit any bias related to self-selection captured via retention or adherence. Using ITT will allow us to determine the overall impact of having the NIPP vs. NIPP+ vs. control in your community, thus better mimicking real-world program implementation and impact. Each survey question will have the option of “Refused to Answer” or “Don’t Know” and will eventually be transformed into missing variables. Prior to that transformation, we will use the proportion of RA or DK responses to understand the sensitivity of the question itself. We will use the standard rule where if fewer than 5% of your observations are missing, the variable will remain in the analysis. If the 5% or more of observations are missing, we will not use the variable to avoid drawing inaccurate inferences. We will not impute missing data.

For the analysis, we will take advantage of the panel to run mixed effects models, allowing us to determine the role of time-variant and invariant individual household, and community characteristics, as well as measures of exposure and integration on key outcome indicators (Table 1 above). In addition, we will use a difference-in-difference analysis (baseline/endline, baseline/sustainability, endline/sustainability) to triangulate findings. To complement these models, the analysis will also leverage exploratory analysis to identify cross-cutting or underlying correlations using principal component analysis. For all of the analysis, we will control for a host of individual-, household-, and community-level characteristics.

The specific models run will depend on the distribution of our key outcome variables. Most of our variables are continuous and therefore we will use either an ordinary least squares, Poisson, or negative binomial regression (depending on the distribution of the variable) to compare across the three treatment arms. For binary variables (such as whether the household reported using improved seeds), we will use a logit model. All analysis will control for the complex survey design. For each key outcome variable, we will run a crude model (meaning only looking at the relationship between the outcome variable and intervention) and an adjusted model (controlling for key household and child demographics such as gender, age, education, and household size). A comprehensive research uptake strategy will be developed alongside stakeholders in Uganda.

#### Analysis of qualitative data

Qualitative data will be analyzed using appropriate software (e.g., NVivo). Patterns emerging from program staff, NIPP and NIPP+ volunteers, and participants will be triangulated to ascertain program process implementation and fidelity.

#### Dissemination policy

The full protocol, all Intellectual Work, and the anonymized participant-level datasets will be made publicly available at the end of the study. The protocol and all Intellectual Work will be posted to USAID’s Development Experience Clearinghouse (DEC) public website. The de-identified dataset will be made available on USAID’s Development Data Library (DDL) public website. Datasets will be submitted to the DDL within thirty (30) calendar days after the publication of the Intellectual Work. The primary audiences for this study are researchers, policymakers, and field-based professionals engaged in nutrition and health in Uganda, the USA, and globally. We will develop a research uptake strategy to link research and learning to policy and practice by using a combination of materials (print and electronic) and events/meetings to market the study and share findings.

We will make every effort to keep the interval between the completion of data collection and the release of the study results to a minimum. In addition to a journal article, we will produce learning briefs, policy briefs, and technical guides, as appropriate based on the findings. Once the study is completed, we will make reports public on Consortium partners’ websites and through the USAID Development Experience Clearinghouse (DEC). To disseminate the findings, we will conduct workshops at the community, national, and district levels in Uganda and present to international audiences at conferences. We will disseminate the final results of the study regardless of the magnitude or direction of effect. According to the grant, the funder may require a preproduction review of the program and public communication materials but has not yet done so. Although the funder may request a preproduction review, they cannot influence the results or final conclusions.

##### ^1^Trial status

Recruitment by invitation began in March 2021. https://www.clinicaltrials.gov/ct2/show/NCT04209569?term=ghosh&draw=2&rank=10

## Supplementary Information

**Additional file 1.** Supplementary Materials: Example High-Energy Recipe.

## Data Availability

The final de-identified dataset will be shared via the Development Experience Clearinghouse (DEC).

## Footnotes

1 While it is possible that the male and female circles may not overlap, the intervention endeavors to identify and recruit households that are assigned to the circles. There are likely to be single-headed households which will be included and will need to be considered within the context of the study implementation.

2 Most inputs are available at low cost and can be sourced in Uganda. We will provide some subsidies but the aim is to get all households to consider these innovations as worthy investments. For example, the hygrometer costs $0.90 per unit and can be used for many years as long as the household/farmer understands the importance and how to use the meter.

3 Hygrometers are battery operated and we have been able to purchase them for about $0.90 per unit through an online reseller. The device is reusable and can detect moisture levels in maize within 15 min and allows farmers and traders to determine whether or not maize is dry enough to be safely stored for later human consumption

4 USAID WASH Plus defines small doable actions as small or baby steps to get closer to the ideal or desired WASH behavior.
