# Supplementary material for: The One Nutrition in Complex Environments (ONCE) study protocol: a cluster-randomized multi-level multi-sectoral intervention to improve nutrition in Uganda
Source: Trials. 2022 Apr 1;23:244. doi: 10.1186/s13063-022-06170-7 (PMC8972632; doi:10.1186/s13063-022-06170-7)
Supplement: Supplementary file 1 — Additional file 1. Supplementary Materials: Example High-Energy Recipe. [file 13063_2022_6170_MOESM1_ESM.docx]

**Supplementary Materials: Example High-Energy Recipe**

To prepare high energy porridge, caregivers may use flour from a range of locally available cereals, such as maize flour, millet flour, sorghum flour. These flours can be substituted depending on mother’s choice and availability to diversify the diet. Ingredients that may be used to enrich the porridge milk, groundnut, sesame, dried fish, eggs and can also be substituted depending on mother’s choice and availability to diversify the diet.

**Recipe 1: Millet, Soy and milk Porridge**

Ingredients

- Millet flour 1 palm / 2-3 tablespoon (90g)
- Soybeans 1/3 palm (30g)
- Milk 1 cup (250ml)
- Cooking Oil 2 teaspoons (10g)
- Sugar 2 teaspoons (16g)
- Water 2 mugs (1000ml)
- Iodized salt 2 pinches (2g)

Preparation

- Wash your hands and your utensils with water and soap and wash thoroughly all of your dried and fresh ingredients.
- Soak the soybeans overnight.
- Remove the testa of soybeans.
- Boil the soybeans until they are tender.
- Mash the soybeans to form a smooth paste.
- Mix the flour with some cold water to form a paste of pouring consistency.
- Add the mixture to the boiling pan of water and stir well.
- Add sugar to taste and leave to boil for 15 minutes.
- Beat the soybean paste into the porridge.
- Add sugar to the porridge and serve.
- Serve the porridge to the child when cool enough and actively feed them.

Age group

- 6-8, 9-11 and 12-23 months (based on the energy and protein requirements of the age group)

Servings

- 1-2 cups for 6-8 months, 2 cups for 9-11 months and 2-3 cups for 12-23 months spread throughout the day.
- Tips/Optional if available during the season: add 2-4 teaspoons of mashed fruits, boiled egg, mashed avocado, to increase the content of protective nutrients and to diversify the diet.

Estimated composition (Nutval, v4.1) per 100g dry matter:

- Energy (Kcal): 772
- Protein (g): 28
- Fat (g): 28
- Vit. A (µg RAE): 205
- Vit. C (mg): 1.8
- Iron (mg): 8.3
- Zinc (mg): 4.8

Health benefits

- Body building foods (proteins) help the body to grow and repair worn out tissues.
- Energy-giving foods (carbohydrates) provide calories for the energy needs of the body, including body metabolism, and also are a source of fibre which is important for good digestion.
- Protective foods (vitamins and minerals) including fruit and vegetables which are very important for protecting the body against infections and the consequences of micronutrient deficiencies.
- Calcium in milk for maintaining strong and healthy bones and teeth, good muscle function.
- Vitamin C, important for boosting the immunity and making Iron in millet bioavailable.
- Provides iron to prevent anaemia.
- Vitamin K for helping wounds to heal.
- Vitamins B for keeping keep skin, eyes and the nervous system healthy.
- Magnesium & selenium for strong bones and teeth.
- Extra energy giving (fats, oils and sugar) consumed in moderation, provide some of our energy and also facilitate the absorption of vitamins.

**Recipe 2: Maize-Millet and milk porridge**

Ingredients

- Maize flour 1 palm / 2-3 tablespoon (90g)
- Millet flour 1 palm / 2-3 tablespoon (90g)
- Milk 1 cup (250ml)
- Cooking Oil 2 teaspoons (10g)
- Sugar 2 teaspoons (16g)
- Water 2 mugs (1000ml)
- Iodized salt 2 pinches (2g)
- Lemon ½ fruit

Preparation

- Wash and dry the pans with clean water and soap before preparing the porridge.
- Wash your hands with clean water and soap.
- Pour the water into the pan and put to boil.
- Blend maize and millet flour together.
- Mix the flour with some cold water to form a paste of pouring consistency.
- Add the mixture to the boiling pan of water and stir well.
- Add sugar to taste and leave to boil for 15 minutes.
- Once the porridge is ready, remove pan from fire, immediately add the oil and the milk and beat well till fully mixed (this is done when the porridge is still hot).
- Now serve the porridge and actively feed the child when porridge is cool enough.

Age group

- 6-8, 9-11 and 12-23 months (based on the energy and protein requirements of the age group)

Servings

- 1-2 cups for 6-8 months, 2 cups for 9-11 months and 2-3 cups for 12-23 months spread throughout the day.
- Tips/Optional if available during the season: add 2-4 teaspoons of mashed fruits, boiled egg, mashed avocado, to increase the content of protective nutrients and to diversify the diet.

Health benefits

- Body building foods (proteins) help the body to grow and repair worn out tissues.
- Energy-giving foods (carbohydrates) provide calories for the energy needs of the body, including body metabolism, and also are a source of fibre which is important for good digestion.
- Protective foods (vitamins and minerals) including fruit and vegetables which are very important for protecting the body against infections and the consequences of micronutrient deficiencies.
- Calcium in milk for maintaining strong and healthy bones and teeth, good muscle function.
- Vitamin C, important for boosting the immunity and making Iron in millet bioavailable.
- Provides iron to prevent anaemia.
- Vitamin K for helping wounds to heal.
- Vitamins B for keeping keep skin, eyes and the nervous system healthy.
- Magnesium and selenium for strong bones & teeth
- Extra energy giving (fats, oils and sugar) consumed in moderation, provide some of our energy and also facilitate the absorption of vitamins.

Supplementary Table 1: Time schedule of enrollment, interventions, and survey rounds

| Activity | Timeframe |
| --- | --- |
| Baseline Household Surveys | March 2021 |
| NIPP and NIPP+ interventions implemented on rolling basis | April – October 2021 |
| Endline surveys (following completion of a NIPP or NIPP+ cycle) | July – October 2021 |
| Qualitative Interviews | August-September 2021;  Februay 2022;  August-September 2022 |
| 2-month follow up survey | September – December 2021 |
| 6-month follow up survey | January – April 2022 |
| Sustainability survey | October 2022 |
